# Synthetic Oligopeptides Mimicking γ-Core Regions of Cysteine-Rich Peptides of *Solanum lycopersicum* Possess Antimicrobial Activity against Human and Plant Pathogens 

**DOI:** 10.3390/cimb43030087

**Published:** 2021-09-24

**Authors:** Marina P. Slezina, Ekaterina A. Istomina, Ekaterina V. Kulakovskaya, Tatiana N. Abashina, Tatyana I. Odintsova

**Affiliations:** 1 Vavilov Institute of General Genetics RAS, 119333 Moscow, Russia; omey@list.ru (M.P.S.); mer06@yandex.ru (E.A.I.); 2 Federal Research Center, Pushchino Scientific Center for Biological Research of the Russian Academy of Science, Skryabin Institute of Biochemistry and Physiology of Microorganisms RAS, 142290 Pushchino, Russia; ekaterina.kulakovskaya@gmail.com (E.V.K.); tnabashina@gmail.com (T.N.A.)

**Keywords:** plant immunity, cysteine-rich peptides, antimicrobial peptides, γ-core, plant pathogens, human pathogens

## Abstract

Plant cysteine-rich peptides (CRPs) represent a diverse group of molecules involved in different aspects of plant physiology. Antimicrobial peptides, which directly suppress the growth of pathogens, are regarded as promising templates for the development of next-generation pharmaceuticals and ecologically friendly plant disease control agents. Their oligopeptide fragments are even more promising because of their low production costs. The goal of this work was to explore the antimicrobial activity of nine short peptides derived from the γ-core-containing regions of tomato CRPs against important plant and human pathogens. We discovered antimicrobial activity in peptides derived from the defensin-like peptides, snakins, and MEG, which demonstrates the direct involvement of these CRPs in defense reactions in tomato. The CRP-derived short peptides appeared particularly active against the gram-positive bacterium *Clavibacter michiganensis,* which causes bacterial wilt—opening up new possibilities for their use in agriculture to control this dangerous disease. Furthermore, high inhibitory potency of short oligopeptides was demonstrated against the yeast *Cryptococcus neoformans*, which causes serious diseases in humans, making these peptide molecules promising candidates for the development of next-generation pharmaceuticals. Studies of the mode of action of the two most active peptides indicate fungal membrane permeabilization as a mechanism of antimicrobial action.

## 1. Introduction

Plants protect themselves from pathogenic microorganisms by reinforcing structural barriers and producing compounds toxic to invaders. Antimicrobial peptides (AMPs), small polypeptide-based molecules, are the integral constituents of the plant immune system. They provide the first line of defense against an armory of plant-colonizing fungi, bacteria, viruses and pests [1]. Synthesized either constitutively or upon pathogen challenge, AMPs inhibit growth of a wide range of pathogens acting in micromolar concentrations. Plant AMPs are structurally diverse; several families with distinct 3D structures and cysteine signatures have been described [2,3,4]. However, the mode of action of AMPs has been elucidated only for a very limited number of peptides, which mainly belong to the defensin family. Limited data on structure–function relationships in AMPs pose an obstacle to the understanding of the molecular mechanisms underlying plant innate immunity. With the advent of high-throughput sequencing, dozens and even hundreds of AMP-like sequences have been discovered in plant genomes [5,6,7]. Whole transcriptome analysis showed that complex arrays of AMPs are involved in response to pathogens and resistance-inducing agents [8,9,10,11]. Both pathogens and elicitors trigger expression of different sets of AMP-like genes, which prevent or impede colonization of plant tissues in combination with other antimicrobial proteins and secondary metabolites. In addition to AMPs, environmental stress affects expression of other cysteine-rich peptides (CRPs) [11], whose role in the immune response is less well understood.

In our previous studies, we explored transcriptional changes in CRP genes in *Solanum lycopersicum* L. plants infected with *Fusarium oxysporum* strain F37 and treated with biogenic elicitors isolated from the non-pathogenic FS-94 strain of *F. sambucinum*. We identified CRP genes which were up-regulated by the pathogen and the elicitors [11]; they encoded defensin-like (DEFL) peptides, snakins, non-specific lipid-transfer proteins (nsLTPs) and MEG (Maternally Expressed Gene) peptides. In this work, nine CRP peptides discovered in tomato transcriptomes were selected for functional studies. Short oligopeptides corresponding to the γ-core motifs of the selected CRPs, which were postulated to play an important role in antimicrobial activity, were synthesized and assayed against a panel of plant and human pathogens including yeasts, bacteria and filamentous fungi. Peptides with strong activity against both plant and human pathogens were discovered. For the first time, regions in snakin and MEG molecules that contribute to antimicrobial activity were identified. In addition to providing an insight into the structural determinants of antimicrobial activity in AMPs, short peptides derived from natural defense molecules present promising templates for designing novel plant disease control agents and pharmaceuticals. Plant diseases caused by pathogenic microorganisms are responsible for huge losses of production in agriculture. Pathogens also reduce the quality and safety of agricultural products. Pesticides and antibiotics used to control diseases have a negative impact on the environment and human health, stimulating the search for novel antimicrobial agents. AMPs are promising candidates for the development of novel biopesticides due to their broad antimicrobial spectrum, activity against multidrug resistant pathogens, low toxicity and biodegradability. However, they are often produced in low amounts in plants, impeding their exploitation in disease control. In contrast, short plant AMP-derived peptides are ideal candidates for practical use in agriculture and medicine due to low production costs. Therefore, shortening of natural AMP sequences is a promising strategy for producing novel highly active low-cost antimicrobials. This approach is especially effective if the determinants of antimicrobial activity in AMPs are known. The goal of this work was to investigate the antimicrobial properties of short peptides derived from the γ-core motifs of tomato cysteine-rich peptides belonging to different families against important plant and human pathogens, in order to shed light on their mode of action, with the prospect for their practical use in agriculture and medicine.

## 2. Materials and Methods

### 2.1. Chemical Synthesis of Short Peptides Derived from Predicted Cysteine-Rich Peptides of Tomato

Solid-phase chemical synthesis using Fmoc chemistry was used to produce peptides γ_58-74_SlDEFL2, γ_58-74_SlDEFL4, γ_48-65_SlSN2, γ_89-106_SlSN9, γ_47-64_SlSN10, γ_56-72_SlLTPg2.4, γ_53-69_SlLTPg2.5, γ_70-86_SlLTPg2.8 and γ_92-104_SlMEG2 corresponding to the γ-core regions of 9 predicted tomato CRPs belonging to the DEFL, snakin, nsLTP and MEG families (Elabscience Biotechnology Inc., Wuhan, China). The synthetic peptides were purified by RP–HPLC. The identity of the synthesized peptides, to ensure they had the required sequences, was confirmed by mass spectrometry.

The following characteristics of the synthesized peptides were calculated using the ExPASy ProtParam tool [12]: molecular weight, isoelectric point (pI), aliphatic index, grand average of hydropathicity (GRAVY) index, and net charge at pH 7. The Boman potential protein interaction index was computed using APD3 [13].

### 2.2. MALDI–TOF MS

The molecular masses of the synthetic peptides were determined by MALDI–TOF MS on an Ultraflex MALDI–TOF mass spectrometer (Bruker Daltonics, Bremen, Germany) in a linear or reflector positive ion mode using α-cyano-4-hydroxycinnamic acid as a matrix.

### 2.3. 3D Structure Modeling

The 3D structure of the synthesized peptides was de novo modeled using PEP-FOLD3 [14]. The best representative models were chosen based on the lowest sOPEP values provided by PEP-FOLD3.

### 2.4. Antimicrobial Assays

#### 2.4.1. Determination of the Antimicrobial Activity of Synthetic Peptides against Yeasts and Bacteria

The antimicrobial activity of synthetic peptides was tested against the yeasts *Cryptococcus neoformans* VKM Y-2755 and *Candida albicans* VKM Y-2994, as well as against the bacteria *Pectobacterium carotovorum* subsp. *carotovorum* VKM B-1247, *Pseudomonas savastanoi* pv. *savastanoi* VKM B-1546 and *Clavibacter michiganensis* subsp. *michiganensis* VKM Ac-1403. All cultures were obtained from the All-Russian Collection of Microorganisms (VKM). Yeasts were grown on YPD-P medium containing (g/L): glucose–10, peptone–5 and yeast extract–4. Bacterial cultures were grown on medium containing (g/L): yeast extract–1, soya extract–30, aminopeptides (a solution of low-molecular weight peptides and all essential amino acids obtained from the blood of cattle by enzymatic hydrolysis)–60, tryptone–5 and pH 7.2. The microbial cultures were grown on a shaker at 30 °C for 24 h. The optical density of the cultures was measured on a UNICO 1201 (Unico, Dayton, NJ, USA) spectrophotometer at 594 nm. Yeast cultures were diluted 1:10 with water before analysis. For experiments with *P. carotovorum* and *C. michiganensis*, a modified YPD-P medium was used, containing (g/L): glucose–5, peptone–10 and yeast extract–5, and for experiments with *P. savastanoi*, the medium did not contain glucose, and the concentrations of peptone and yeast extract were 20 g/L and 10 g/L, respectively. The antibiotic activity of peptides against yeasts and bacteria was assayed in immunoassay microtiter plates. 10 µL of the tested peptide solution (final concentrations of 10–300 µM) in water, 80 µL of the medium and 10 µL of the microbial suspension were mixed in a well of the microtiter plate and incubated at 30 °C for 24 h; after that, the absorbance of the suspension was measured on an Efos 9305 spectrophotometer (Sapphire, Moscow, Russia) at 594 nm.

#### 2.4.2. Determination of the Antimicrobial Activity of Peptides against Plant Pathogenic Fungi

To study the effect of peptides on spore germination and growth of plant pathogenic fungi, fungal strains from the VKM collection were used: *Fusarium culmorum* VKM F-2303, *F. oxysporum* VKM F-137, *F. solani* VKM F-142, *F. verticillioides* VKM F-670, *Bipolaris sorokiniana* VKM F-4006, and *Botrytis cinerea* VKM F-4549. The fungi were grown on potato dextrose agar at 25 °C for 7–8 days, or for *B. sorokiniana* and *B. cinerea*, for 10–12 days. Spores were washed off from the surface of the mycelia with sterile distilled water and filtered through a sterile filter. The filtrate was centrifuged at 6000 rpm for 15 min. The precipitate was washed three times with sterile distilled water, transferred to Eppendorf tubes, and centrifuged at 10,000 rpm for 2 min. 1 mL of sterile aqueous 20% glycerol solution was added to the spores, and the tubes with spores were stored in a freezer at −20 °C.

Similar to antimicrobial assays with yeasts and bacteria, the effect of peptides on spore germination and fungal growth was studied by measuring the absorbance of the spore suspension in the presence of the peptide in microtiter plates. The wells of a plate were filled with 90 µL of spore suspension in half-strength potato dextrose broth at a concentration of 2000–3000 spores in 100 µL and 10 µL of aqueous solutions of peptides at final concentrations of 10–300 µM. The concentration of spores in the suspension was determined in the Goryaev chamber. The absorbance was recorded at 595 nm on a FilterMax F5 Multi-Mode Microplate Reader (Molecular Devices, San Jose, CA, USA) after 38 h of incubation, for *B. sorokiniana* and *B. cinerea*, after 14, 19, 24, 38, 43, 62 h.

Antimicrobial activity was expressed in IC_50_ values, which denote the concentration required for 50% inhibition of the pathogen growth; this was determined from a graph showing the dependence of antimicrobial activity on peptide concentration.

### 2.5. Statistical Analysis

For each pathogen, experiments on the evaluation of antimicrobial activity of the synthetic peptides were performed with three replicates per treatment. Mean values, standard deviations (SD), and the significance of differences (*p* ≤ 0.05) of the means between treatments and controls (t-test for independent variables) were determined using STATISTICA v. 6.1 software (StatSoft Inc., Tulsa, OK, USA).

### 2.6. Staining of C. albicans Cells with Propidium Iodide

Two peptides, γ_58-74_SlDEFL4 and γ_89-106_SlSN9, at a concentration of 300 µM were chosen for analysis. The cells of *C. albicans* (24 h culture in YPD-P at a cell concentration of 2 × 10^8^/mL) were incubated with peptides at 30 °C for 1 h. After incubation, 1 mL of the cells was stained with 0.03 mM propidium iodide (20 mM stock solution in DMSO was used). The staining proceeded for 15 min at 30 °C. The fluorescence was recorded on an AXIO Imager A1 fluorescence microscope (ZEISS, Göttingen, Germany) using Zeiss filter set 56 HE. The excitation filters 556/25 were placed in the DG5 filter box. The dichroic cube held the beam splitter (DFT 490 + 575) and dual bandpass filter (DBP 512/30 + 630/98) for propidium iodide with excitation at 538 nm and emission at 617 nm.

## 3. Results

### 3.1. Design

**Figure 1:**
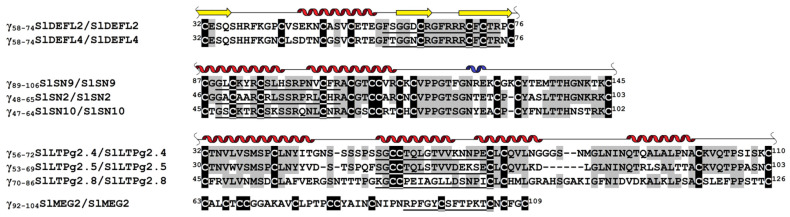
Multiple sequence alignment of the γ-core-containing regions of selected tomato CRPs. Superscript numbers denote their position in the precursor proteins. The sequences of synthesized peptides are underlined. The names of synthetic peptides and their parent CRPs are shown on the left. Cysteine residues are shaded black, and identical amino acids are shaded gray. Secondary structure elements (α- and 3_10_-helices, and β-strands) are shown above the corresponding sequences as helices (α-helix is shown in red, and 3_10_-helix is colored blue) and arrows, respectively. In snakins, the secondary structure was predicted by Porto and Franco [17].

It has been generally acknowledged that the antimicrobial activity of plant AMPs is associated with their γ-core motifs, which are short sequences with a GXCX_3-9_C signature adopting a β-hairpin structure [15,16]. Tomato CRPs discovered in transcriptomes of plants infected by *F. oxysporum* and treated with *F. sambucinum* elicitors [11] were screened for the presence of γ-core sequences. The γ-core motifs were discovered in DEFLs, snakins, nsLTPs and MEG peptides. Among the γ-core-containing CRPs, we selected those peptides that were involved in defense responses to infection or elicitor-mediated induced resistance (IR). The selected CRPs included 2 DEFLs, 3 snakins, 3 nsLTPs and 1 MEG. Nine short peptides encompassing the γ-core region of these CRPs were produced by solid-phase synthesis and assayed against a panel of pathogenic microorganisms. The synthetic peptide sequences and their position in the amino acid sequences of the CRP precursors are shown in Figure 1.

#### 3.1.1. DEFLs

Nine DEFL sequences were discovered in transcriptomes of tomato plants infected with *F. oxysporum*, treated with *F. sambucinum* elicitors and infected after elicitor treatment [11]. Of them, only three DEFL genes were responsive to *F. oxysporum* infection or elicitor treatment. Two of them, SlDEFL2 and SlDEFL4, were selected for the synthesis of γ-cores. The SlDEFL2 gene was up-regulated by *F. sambucinum* elicitors, while expression of the SlDEFL4 gene was induced by *F. oxysporum* infection. The γ-core regions GXCXnC were synthesized together with the flanking residues X_3_GXCX_6_CXCX_2_, because it was shown that such extended γ-core peptides possess higher antimicrobial activity than classical γ-cores [16]. The synthetic DEFL-derived peptides were named γ_58-74_SlDEFL2 and γ_58-74_SlDEFL4. They were highly similar, differing in only two amino acid residues at positions 2 (S/T) and 5 (D/N) (Figure 1).

#### 3.1.2. Snakins

In tomato transcriptomes, we found thirteen sequences of snakin precursors [11]. Of them, eleven snakin genes were differentially expressed in infected and/or elicitor-treated plants. Of the differentially expressed genes, only three genes encoding SlSN2, SlSN9 and SlSN10 possessed a γ-core motif. The SlSN2 gene was particularly highly expressed in all transcriptomes. Infection down-regulated SlSN2 and SlSN9 genes; however, in IR-displaying plants (challenged with *F. oxysporum* after elicitor treatment), the expression levels of all three genes were significantly enhanced, pointing to their important roles in induced resistance [11]. The peptides with the GXCX_11_CX_2_ motif were chosen for chemical synthesis (Figure 1). The amino acid sequences of snakin-derived peptides were more variable than those of the SlDEFLs.

#### 3.1.3. nsLTPs

In tomato transcriptomes, we identified 44 sequences of putative nsLTPs. Only four polypeptides possessed the γ-core motif. The γ-core-containing fragments of SlLTPg2.4, 2.5 and 2.8 with the XGXCX_12_C motif were selected for chemical synthesis (Figure 1). The expression level of SlLTP2.8 did not change in infected or elicitor-treated tomato plants. In contrast, the SlLTP2.4 gene was up-regulated by *F. oxysporum* inoculation and in IR-displaying plants, while SlLTP2.5 was up-regulated by the elicitors and inoculation of elicitor-pretreated plants. The sequences of γ_56-72_SlLTPg2.4 and γ_53-69_SlLTPg2.5 were similar, while the sequence of γ_70-86_SlLTPg2.8 was less conserved.

#### 3.1.4. MEG

Of the two MEG-like peptides, both possessed the γ-core region, and both genes were suppressed either by the pathogen (*SlMEG2*) or by the elicitors (*SlMEG1*). Since the role of MEG polypeptides in the immune response remains enigmatic, we chose a SlMEG2 γ-core-containing fragment with the X_3_GXCX_6_C motif for chemical synthesis (Figure 1).

### 3.2. Physicochemical Properties of Short CRP-Derived Synthetic Peptides

The calculated physicochemical properties of synthesized peptides, which are important for antimicrobial activity, are presented in Table 1.

The peptides contain 13 to 18 amino acid residues. Their molecular weights are in the range of 1506.76 to 2139.54 Da. The isoelectric points of the peptides vary from 4.14 in γ_53-69_SlLTPg2.5 to 11.40 in γ_58-74_SlDEFL4 and γ_48-65_SlSN2. In the DEFL-, snakin- and MEG-derived peptides, positively charged residues prevail, resulting in a high positive charge at neutral pH. The highest positive charge of +5 in γ_58-74_SlDEFL4, γ_48-65_SlSN2 and γ_47-64_SlSN10 is due to the presence of five positively charged residues (Arg or Lys). In γ_89-106_SlSN9, there are only four positively charged residues, providing the net charge value of +4, and in γ_58-74_SlDEFL2, there are five positively charged residues and a single negatively charged residue, also resulting in the net charge of +4. The MEG-derived peptide γ_92-104_SlMEG2 is also positively charged, but its charge is lower (+2) than those of DEFL- and snakin-derived peptides. In contrast, in the LTP-derived peptides, the number of positively charged residues is either identical to that of negatively charged residues (γ_56-72_SlLTPg2.4), or even lower. The γ_56-72_SlLTPg2.4 peptide is not charged, and two other LTP-derived peptides are negatively charged (−2 for γ_53-69_SlLTPg2.5 and −1 for γ_70-86_SlLTPg2.8). It has been acknowledged that a positive charge facilitates interactions of peptides with negatively charged membranes of pathogenic microorganisms [18]. However, anionic peptides exhibiting antimicrobial properties have also been described [19]. The aliphatic index, which correlates with thermal stability, is 0 for DEFL- and MEG-derived peptides due to the absence of Val, Ala, Ile and Leu residues, and varies from 21 to 97 for other peptides, being the highest for γ_70-86_SlLTPg2.8. The GRAVY index, reflecting peptide hydrophobicity, is negative for all peptides except for γ_70-86_SlLTPg2.8. A negative GRAVY index characterizes peptides that are more hydrophilic and better dissolve in water than those with a positive GRAVY index. The Boman index, reflecting protein-binding potential, varies from 0.22 in γ_70-86_SlLTPg2.8 to 4.5 in γ_47-64_SlSN10. Peptides that have a Boman index above 2.48 have a high protein-binding potential. All DEFL- and snakin-derived peptides have a Boman index higher than 2.48.

### 3.3. Modeling of the 3D Structures of Synthetic Peptides

**Figure 2:**
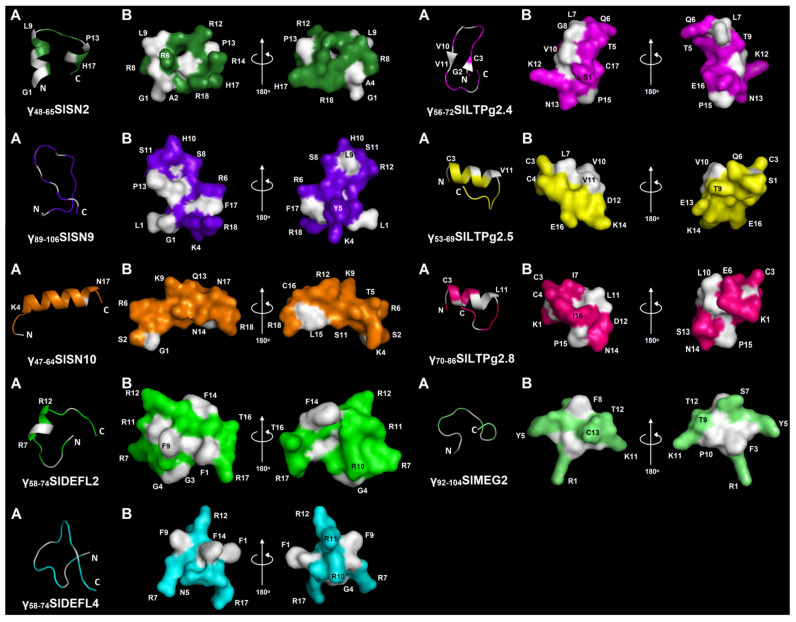
3D molecular modeling of synthetic peptides: (**A**) spatial structure (ribbon representation); (**B**) surface structure. N- and C-termini are marked with N and C, respectively. Non-polar residues are shown in white, polar residues are colored. Modeling was performed using PEP-FOLD3 [14].

The 3D structures of synthetic peptides were modeled with PEP-FOLD 3 [14] (Figure 2). Five peptides contained only α-helices: γ_48-65_SlSN2, γ_47-64_SlSN10, γ_58-74_SlDEFL2, γ_53-69_SlLTPg2.5 and γ_70-86_SlLTPg2.8. A single α-helical region was predicted in γ_47-64_SlSN10, γ_58-74_SlDEFL2, γ_53-69_SlLTPg2.5, γ_70-86_SlLTPg2.8, while two α-helices were predicted in γ_48-65_SlSN2. In γ_58-74_SlDEFL2, the helical region is located in the middle of the peptide, while in γ_53-69_SlLTPg2.5 and γ_70-86_SlLTPg2.8, it is in the N-terminal region of the molecule, followed by an unstructured C-terminal region. In γ_47-64_SlSN10, the α-helical region was predicted to occupy most of the peptide’s molecule. Most α-helical peptides are amphipathic with hydrophobic and polar residues located on opposite sides of the helix. Two antiparallel β-strands were predicted in γ_56-72_SlLTPg2.4. The remaining three peptides (γ_89-106_SlSN9, γ_58-74_SlDEFL4 and γ_92-104_SlMEG2) were predicted to be in a random coil conformation.

### 3.4. Antimicrobial Activity of Synthetic Peptides

**Figure 3:**
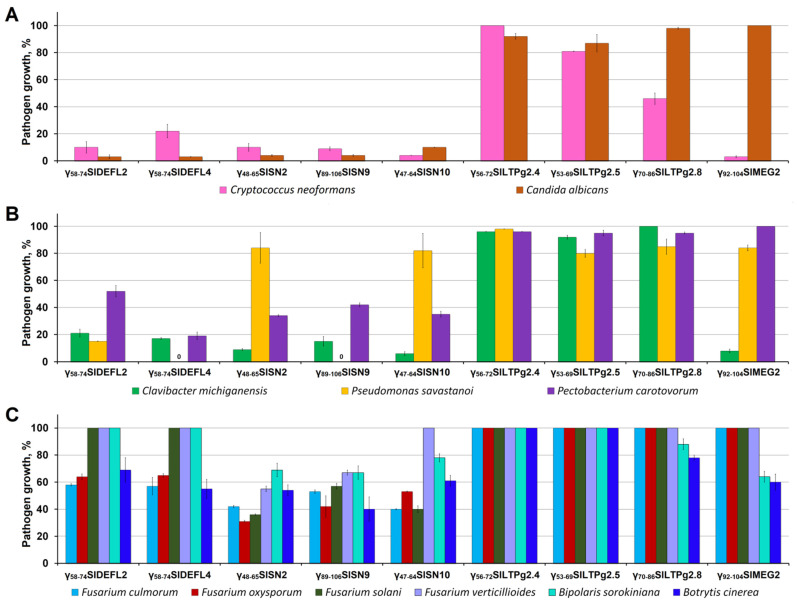
Growth of pathogens in the presence of synthetic peptides (300 μM): (**A**) yeasts, (**B**) bacteria, (**C**) plant pathogenic fungi. Bars represent mean ± SD of growth compared to control (pathogen growth in the absence of peptide).

The peptides were initially screened against a number of pathogenic microorganisms at a concentration of 300 μM (Figure 3). All peptides showed antimicrobial activity. The degree of inhibition depended both on the peptide and the pathogen species. Both DEFL-derived peptides demonstrated high activity against yeasts and bacteria. It is of interest that although these peptides differed only in a pair of amino acid residues, resulting in a higher net charge of γ_58-74_SlDEFL4, their antimicrobial properties differed. Of the pathogenic yeasts, *C. albicans* was more sensitive to the DEFL-derived peptides than *C. neoformans*. γ_58-74_SlDEFL2 was more potent than γ_58-74_SlDEFL4. Inhibition of *C. albicans* amounted to 97% for both DEFL-derived peptides, while that of *C. neoformans* amounted to 90% for γ_58-74_SlDEFL2. Both gram-positive (*C. michiganensis*) and gram-negative bacteria (*P. savastanoi* and *P. carotovorum*) were sensitive to the DEFL-derived peptides. A positive correlation between the net charge and inhibitory activity against bacteria was observed: γ_58-74_SlDEFL4 was more active than γ_58-74_SlDEFL2 (Figure 3B). The degree of inhibition varied from 100% for γ_58-74_SlDEFL4 against *P.* *savastanoi* to 48% for γ_58-74_SlDEFL2 against *P. carotovorum,* while γ_58-74_SlDEFL2 was less active against all tested bacterial species (Figure 3B). In contrast to bacteria and yeasts, the activity of both DEFL-derived peptides against *Fusarium* species was similar and much lower (Figure 3C). *F. verticillioides* and *F. solani* were not inhibited by γ_58-74_SlDEFL2 and γ_58-74_SlDEFL4, while *F. culmorum* was suppressed by 42–43% and *F. oxysporum* by 35–36% by both DEFL-derived peptides. Similar to *F. verticillioides* and *F. solani*, *B sorokiniana* was insensitive to γ_58-74_SlDEFL2 and γ_58-74_SlDEFL4; conversely, *B. cinerea* was inhibited by 31–45%.

Similar to the DEFL-derived peptides, all three snakin-derived peptides showed variations in activity against diverse pathogens (Figure 3). Again, the highest activity was observed against yeasts: *C. albicans* and *C. neoformans*. *C. michiganensis* and *P. carotovorum* were also effectively inhibited. The snakin-derived peptides were even more potent than DEFL-derived peptides against *C. michiganensis*, whose growth was suppressed by 85–94%. γ_89-106_SlSN9 completely inhibited growth of *P.* *savastanoi*, while the other two snakin-derived peptides were only weakly active against this pathogen. In contrast to the DEFL-derived peptides, the snakin-derived peptides were more active against *Fusarium* species and *B. sorokiniana*. All *Fusarium* species, with one exception, were suppressed by the snakin-derived peptides. Only *F. verticillioides* was not sensitive to γ_47-64_SlSN10. The suppression of *Fusarium* species varied from 33% to 69%. The inhibition of *B. sorokininana* growth was rather weak, ranging from 22% to 31%. *B. cinerea* was more efficiently inhibited than *B. sorokiniana*. The degree of *B. cinerea* inhibition changed from 39% to 60%.

All three LTP-derived peptides were much less active than the DEFL- and snakin-derived peptides, which correlated with their neutral or acidic character (Figure 3). Only γ_70-86_SlLTPg2.8 suppressed growth of *C. neoformans* by 54%. The inhibition of *C. albicans* did not exceed 13%, and the suppression of bacterial species was below 20%. γ_56-72_SlLTPg2.4 and γ_53-69_SlLTPg2.5 were inactive against all tested *Fusarium* species, *B. sorokiniana* and *B. cinerea.*

The MEG-derived peptide γ_92-104_SlMEG2 inhibited the growth of *C. neoformans* and *C. michiganensis* with high efficiency (Figure 3). The activity against *B. sorokiniana* and *B. cinerea* was 36–40%. *Fusarium* species were insensitive to γ_92-104_SlMEG2.

**Figure 4:**
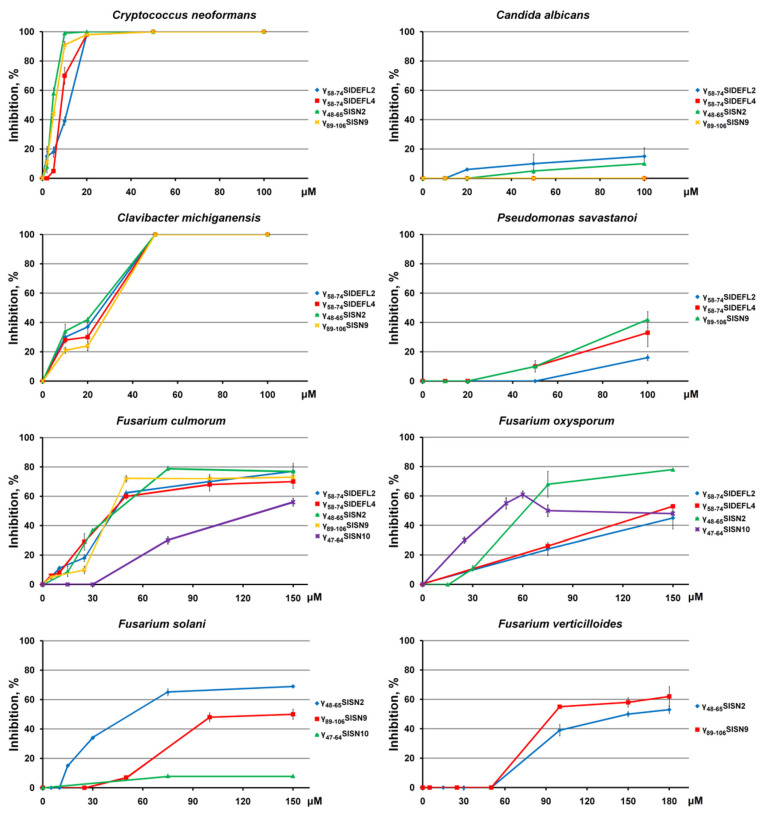
Inhibition curves of pathogenic microorganisms in the presence of different concentrations of tomato CRP-derived peptides (relative to control, %). Bacteria and yeasts were incubated with peptides for 24 h, and plant pathogenic fungi were treated for 38 h. Error bars represent the SD of technical triplicates.

For the DEFL- and snakin-derived peptides, the inhibition dynamics at lower peptide concentrations were determined (Figure 4), and the IC_50_ values were calculated (Table 2). The maximum tested peptide concentration was 100 μM for yeasts and bacteria, 150 μM for *F. culmorum, F. solani* and *F. oxysporum* and 180 μM for *F. verticillioides*. All tested DEFL- and snakin-derived peptides displayed the highest activity against *C. neoformans,* and γ_48-65_SlSN2 and γ_89-106_SlSN9 were more potent inhibitors of the pathogen than γ_58-74_SlDEFL2 and γ_58-74_SlDEFL4. *C albicans* was much more tolerant to the peptides; the maximum inhibition level did not exceed 15% at 100 μM. Of the bacterial pathogens, *C. michiganensis* proved highly sensitive to the peptides (Figure 4, Table 2). *P. savastanoi* was also inhibited by the peptides—however, at higher concentrations.

Of Fusarium species, *F. culmorum* and *F. oxysporum* were the most sensitive species. 79% inhibition of *F. culmorum* growth was achieved with γ_48-65_SlSN2 at a concentration of 75 μM, and 72% inhibition with γ_89-106_SlSN9 at a concentration of 50 μM, γ_47-64_SlSN10 was the least efficient inhibitor of *F. culmorum*.

The activity of both DEFL-derived peptides against *F. culmorum* was similar to that of γ_48-65_SlSN2 and γ_89-106_SlSN9. γ_48-65_SlSN2 showed the highest activity against *F. oxysporum*. On the whole, the snakin-derived peptides γ_48-65_SlSN2 and γ_47-64_SlSN10 were more potent inhibitors of *F. oxysporum* than the DEFL-derived peptides.

*F. solani* and *F. verticillioides* were also efficiently suppressed by the snakin-derived peptides γ_48-65_SlSN2 and γ_89-106_SlSN9. γ_48-65_SlSN2 was more efficient against *F. solani*, with a maximum inhibition of 69% at 150 μM, while γ_89-106_SlSN9 was more potent against *F. verticillioides,* with a maximum inhibition of 62% at 180 μM.

**Figure 5:**
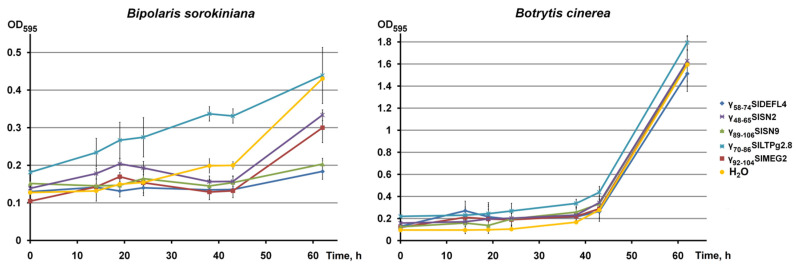
*B. sorokiniana* and *B. cinerea* growth dynamics in the presence of synthetic peptides at a concentration of 60 μM (control−H_2_O). Error bars represent the SD of technical triplicates.

The growth dynamics of *B. sorokiniana* and *B. cinerea* were studied at a peptide concentration of 60 μM (Figure 5). At this concentration, *B. cinerea* was insensitive to all tested peptides (γ_58-74_SlDEFL4, γ_48-65_SlSN2, γ_89-106_SlSN9, γ_70-86_SlLTPg2.8 and γ_92-104_SlMEG2). Conversely, all peptides except γ_70-86_SlLTPg2.8 caused significant growth suppression of *B. sorokiniana*; this effect was observed after 38 h of spore incubation with all peptides. γ_89-106_SlSN9 and γ_58-74_SlDEFL4 were more active than γ_48-65_SlSN2 and γ_92-104_SlMEG2.

**Figure 6:**
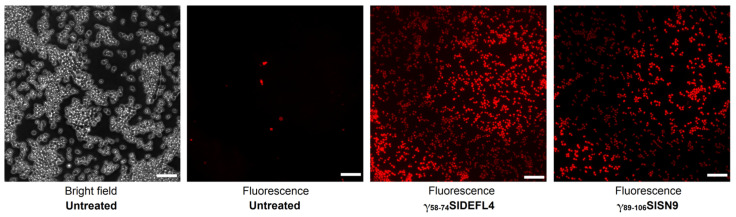
Images of *C. albicans* cells incubated in the presence of 300 μM of γ_58-74_SlDEFL4 and γ_89-106_SlSN9 peptides for 1 h and stained with propidium iodide. Untreated *C. albicans* cells were used as negative control. Scale bar = 20 μm.

To study the mode of action of synthetic peptides, two highly active peptides, γ_58-74_SlDEFL4 and γ_89-106_SlSN9, were selected. Staining of *C. albicans* cells with propidium iodide in the presence of these peptides was carried out and analyzed using fluorescence microscopy (Figure 6). Both peptides induced accumulation of the fluorescent dye inside the cells, which indicated permeabilization of the fungal membranes as a mechanism of both peptides’ action.

## 4. Discussion

The necessity of developing novel antimicrobials for medicine and agriculture stimulates the search for innovative molecules devoid of the disadvantages of conventional antibiotics, such as resistance development and negative ecological impact. Plant CRPs, including AMPs and other peptides with defense functions, represent a valuable pool of natural antibiotics with high potential for practical applications. Short CRP-derived peptides are particularly promising for the development of next-generation antimicrobials due to low production costs.

The objective of this work was to study the antimicrobial activity of nine short peptides derived from CRPs discovered in the transcriptomes of tomato plants infected with *F. oxysporum* and treated with biogenic resistance inducers, which were responsive to the infection and/or elicitors. A panel of eleven fungi and bacteria pathogenic to plants and humans were used in the antimicrobial assays.

For chemical synthesis, in tomato DEFLs, snakins, nsLTPs and MEG peptides, we selected short sequences with a γ-core motif GXCX_3-9_C [15]. This motif was postulated to be ubiquitous in AMPs and vital for their antimicrobial properties [15,16]. In defensins, it has been shown that this short sequence is positively charged and adopts a β-hairpin structure connecting and partially encompassing the β2 and β3 strands in the defensin 3D structure. We synthesized two peptides, γ_58-74_SlDEFL2 and γ_58-74_SlDEFL4, comprising the γ-core sequence with seven additional amino acid residues—three from the N-terminus and four from the C-terminus (Figure 1, Table 1). The spatial structure of snakins has not been resolved so far. However, molecular modeling predicts that the snakin molecule consists of two long α-helices connected by three disulfide bonds, while three remaining disulfides connect the second α-helix to the mostly unstructured C-terminal half of the molecule containing one short 3_10_-helix [17]. The “extended” γ-core motifs (peptides γ_48-65_SlSN2, γ_89-106_SlSN9 and γ_47-64_SlSN10) chosen for synthesis in snakins comprise most of the first α-helix, a loop connecting both α-helices and half of the second α-helix (Figure 1). The fold of nsLTPs includes four or five parallel α-helices forming a tunnel-like cavity for lipid binding [20]. The nsLTP-derived peptides selected for synthesis—γ_56-72_SlLTPg2.4, γ_53-69_SlLTPg2.5 and γ_70-86_SlLTPg2.8—possess a γ-core signature with an additional N-terminal amino acid residue, which encompasses the second α-helix, the loop connecting the second and the third α-helix, and the beginning of the third helix. The 3D structure of MEG peptides is unknown, so the location of the γ-core motif with three additional N-terminal amino acid residues chosen for chemical synthesis (γ_92-104_SlMEG2) in the spatial structure of SlMEG2 remains unclear. All synthetic peptides were positively charged, except for the nsLTP-derived peptides, which were either negatively charged (γ_53-69_SlLTPg2.5 and γ_70-86_SlLTPg2.8) or neutral (γ_56-72_SlLTPg2.4).

In our work, eleven pathogens, including yeast-like fungi pathogenic to humans, as well as plant pathogenic bacteria and fungi, were used in antimicrobial assays. We showed that all tested peptides except the nsLTP-derived peptides were active against at least several pathogens. This activity depended on both the peptide and the pathogen tested. γ_56-72_SlLTPg2.4 and γ_53-69_SlLTPg2.5 were virtually inactive, which correlates with their anionic or neutral nature. The positively charged DEFL- and snakin-derived peptides displayed broad-spectrum activity, while the MEG-derived peptide showed narrow-range activity. It is of interest that the synthetic peptides displayed higher activity against yeasts and plant pathogenic bacteria than against plant pathogenic fungi.

Of the yeasts, two species were taken for antimicrobial assays: *Cryptococcus neoformans* and *Candida albicans*. *Candida* spp. (Ascomycota) affect immunocompromised people, causing candidiasis. In patients with systemic candidiasis due to *C. albicans*, the mortality rate can be as high as 40% [21]. Another tested human pathogenic yeast-like fungus *C. neoformans* is also usually associated with infections in immunocompromised patients, causing life-threatening fungal pneumonia and meningitis [22]. Globally, approximately 1 million cases of cryptococcosis are reported each year, resulting in approximately 625,000 deaths [23].

Our results show that at a high peptide concentration (300 µM) all DEFL- and snakin-derived peptides effectively inhibited both *C. neoformans* and *C. albicans* (Figure 3). The MEG-derived peptide was also highly active against *C. neoformans.* At lower peptide concentrations (≤100 µM), the high inhibitory activity of the peptides was retained against *C. neoformans* (Figure 4). The snakin-derived peptides were even more potent inhibitors of *C. neoformans* than DEFL-derived peptides, making these peptides valuable templates for the development of novel pharmaceuticals to combat *C. neoformans*-associated diseases.

Of the plant pathogenic bacteria, three species were used in antibacterial assays: *Clavibacter michiganensis* subsp. *michiganensis, Pseudomonas savastanoi* subsp. *savastanoi* and *Pectobacterium carotovorum* subsp. *carotovorum.*

*C. michiganensis* is a gram-positive actinomycete plant pathogenic bacterium displaying a biotrophic lifestyle [24]. It causes systemic vascular infections leading to wilt, leaf necrosis, stem cankers and, ultimately, the death of their host plants. *C. michiganensis* subsp. *michiganensis* is the causal agent of bacterial wilt and canker in tomato and is one of the most important bacterial pathogens of tomato. Bacterial canker causes severe yield losses leading to 46% to 93% plant death and ~50% decreases in average fruit weight during severe epidemics.

*P. savastanoi* is a gram-negative, aerobic plant pathogenic bacterium that infects a variety of plants. *P. savastanoi* pv. *savastanoi* causes a disease named olive knot in cultivated and wild olive and some other woody plants. Olive knot can cause severe damage to olive trees in most olive-growing regions [25].

*P. carotovorum* is a necrotrophic gram-negative plant pathogenic bacterium with a diverse host range, which infects more than 100 agriculturally important crops and ornamental plants. It is one of the top ten plant pathogenic bacteria based on scientific and economic importance [26]. The diseases caused by *P. carotovorum* in plants, such as soft rot, wilt and blackleg, lead to important economic losses worldwide [27]. *P. carotovorum* subsp. *carotovorum* is the most common pathogen causing soft rot, infecting plants in at least 16 dicotyledonous and 11 monocotyledonous families [27].

Antimicrobial assays with plant pathogenic bacteria showed that at high peptide concentrations, all DEFL-, snakin- and MEG-derived peptides were highly active against the gram-positive bacterium *C. michiganensis* (Figure 3). γ_58-74_SlDEFL2 also effectively inhibited the gram-negative bacterium *P. savastanoi*, while γ_58-74_SlDEFL4 and γ_89-106_SlSN9 completely inhibited growth of this bacterium. γ_58-74_SlDEFL4 also efficiently suppressed growth of the gram-negative bacterium *P. carotovorum*. At lower peptide concentrations, high inhibitory activity remained against *C. michiganensis* (Figure 4). Both DEFL- and snakin-derived peptides were highly active against this bacterium, which makes it possible to use these peptides as novel antimicrobial agents to control bacterial wilt and canker of tomato.

Of the plant pathogenic fungi, several *Fusarium* species, *Bipolaris sorokiniana* and *Botrytis cinerea* were used in antifungal assays. *Fusarium* fungi (Ascomycota) belong to the hemibiotrophic pathogens. Their special place among other pathogens is associated with their so-called trans-kingdom pathogenicity, which is the ability to infect and cause diseases in humans, animals and plants using fundamentally distinct infection strategies [28]. *Fusarium* fungi cause diseases of economically important crops by reducing yields and decreasing product quality due to the production of mycotoxins toxic to humans and cattle. *F. oxysporum* is a ubiquitous soilborne pathogen which causes vascular wilt in more than 100 plant species. It ranks fifth among the top ten fungal plant pathogens [28]. In humans, *Fusarium* fungi affect immunocompromised patients, causing both superficial (keratitis and onychomycosis), locally invasive and disseminated diseases [29].

Of *Fusarium* species, *F. culmorum* and *F. oxysporum* were the species most sensitive to the peptides. The snakin- and DEFL-derived peptides proved highly effective in inhibiting *Fusarium* fungi. The snakin-derived peptides γ_48-65_SlSN2 and γ_89-106_SlSN9 displayed the highest activity against *F. culmorum* (Figure 4). The activity of both DEFL-derived peptides against *F. culmorum* was similar to that of γ_48-65_SlSN2 and γ_89-106_SlSN9. γ_48-65_SlSN2 was the most active peptide against *F. oxysporum.* The γ_48-65_SlSN2 and γ_47-64_SlSN10 peptides were more potent inhibitors of *F. oxysporum* than the DEFL-derived peptides. *F. solani* was also efficiently inhibited by the snakin-derived peptides γ_48-65_SlSN2 and γ_89-106_SlSN9. Thus, the snakin-derived peptides, as well as DEFL-derived peptides, are promising molecules for the development of novel biogenic agents to control fusarioses.

Besides *Fusarium* fungi, two other plant pathogens, *B. sorokiniana* and *B. cinerea,* were included in our antifungal assays. *B. sorokiniana* is a hemi-biotrophic ascomycete fungus with a wide host range in the Poaceae family, which can infect and cause disease on different plant tissues. *B. sorokiniana* is of greatest economic importance as the causal agent of the common root rot and spot blotch seedling diseases of barley and wheat [30]. Grain yield losses due to common root rot and seedling blight amount to 10–20%. Spot blotch can cause significant losses (15–25%) in warm regions [31].

Our results show that at high peptide concentrations (300 μM) *B. sorokiniana* is suppressed by γ_48-65_SlSN2, γ_89-106_SlSN9 and γ_92-104_SlMEG2; however, the degree of inhibition is not very high. At lower peptide concentrations (60 μM) this pathogen was effectively suppressed by all tested peptides except γ_70-86_SlLTPg2.8.

*B. cinerea* is a broad-host-range, necrotrophic plant pathogen which belongs to Ascomycota; it infects mainly dicotyledonous plants [32]. The most common disease caused by *B. cinerea* is gray mold. This disease is widespread around the world and results in serious economic losses due to pre- and postharvest crop losses.

In our antifungal assays, *B. cinerea,* at high peptide concentrations of 300 μM, was efficiently inhibited by γ_89-106_SlSN9 and two other snakin-derived peptides; however, their inhibitory activity against this pathogen was lower. Thus, the tested peptides can serve as templates for the development of novel antifungal agents to control diseases caused by *B. sorokiniana* and *B. cinerea*.

Summarizing the results obtained, we can conclude that the γ-core-containing regions in SlDEFLs (γ_58-74_SlDEFL2 and γ_58-74_SlDEFL4 peptides), which conform to the definition of a classical γ-core (GXCX_3-9_C signature, β-hairpin conformation and a positive charge [15]) possess antimicrobial activity against a wide range of pathogens. The highest activity was observed against yeasts and plant pathogenic bacteria, especially against the yeast *C. neoformans* and the gram-positive bacterium *C. michiganensis*. Of *Fusarium* species, *F. culmorum* appeared to be the most sensitive species. The antimicrobial properties of γ-cores of defensins were reported earlier in a number of studies [16,33,34,35,36,37,38,39,40,41,42,43,44]. However, in our study, for the first time, a broad panel of plant and human pathogens, including fungi and bacteria, was simultaneously assayed and shown to be sensitive to the DEFL-derived γ-core-containing peptides. It is worth noting that γ_58-74_SlDEFL2 and γ_58-74_SlDEFL4 possess a RGFRRR sequence within the γ-core, which occurs in a number of defensins from different plant families. This motif alone was shown to be active against *F. graminearum*, providing entry into fungal cell walls [16,45]. We discovered that although γ_58-74_SlDEFL2 and γ_58-74_SlDEFL4 differ by only two amino acid residues, affecting the charge of the peptide (γ_58-74_SlDEFL4 has a higher net positive charge than γ_58-74_SlDEFL2), their antimicrobial activity against some tested pathogens differed. For the plant pathogenic bacteria and the yeast *C. neoformans*, we observed a positive correlation between the peptide’s charge and the degree of inhibition at high peptide concentrations. Taking into account that one of the two amino acid substitutions in γ_58-74_SlDEFL2 and γ_58-74_SlDEFL4 is located in the neighboring region of the classical γ-core region, we may conclude that adjacent regions also contribute to the antimicrobial activity of the peptide against these pathogens.

Our results for the first time demonstrate that the γ-core-containing regions in snakins possess potent antimicrobial activity, although the length of the loop between the second and the last cysteine residues is longer than in “classical” γ-core motifs. The 3D structure of snakins has not been resolved so far. However, molecular modeling suggests that the snakin molecule resembles α-hairpinins, although with longer α-helices [17]; so, the β-hairpin conformation of the γ-core motif in snakins is highly improbable, although the high net positive charge required for the classical γ-cores is preserved. Nevertheless, our results clearly demonstrate that among all tested peptides, the snakin-derived peptides possess the highest inhibitory activity and have the broadest antimicrobial specificity. Thus, the γ-core-containing sequence has been identified as a determinant of antimicrobial activity in snakins. However, we cannot exclude that other parts of the molecule also contribute to the antimicrobial properties of snakins. In this regard, potato SN1, which displays potent antifungal activity but does not have a typical γ-core sequence, should be mentioned [46].

Analysis of the antimicrobial activity of the nsLTP-derived peptides showed that they are either inactive or exhibit poor antimicrobial activity against the tested pathogens. This correlates with the negative charge or the absence of charge in the nsLTP-derived peptides. The lack of antimicrobial activity in the nsLTP-derived peptides supports our hypothesis that their parent molecules participate in the defense response of tomato to infection and resistance inducers, not as antimicrobial agents, but as signaling molecules activating the immune reactions.

The role of MEG peptides in plant physiology is poorly understood. The *MEG* gene is expressed only in the basal layer of maize endosperm transport cells [47]. It was suggested that in maize, the MEG2 peptide acts as a structural or defense protein. A regulatory role in the transport of nutrients to developing embryos was also hypothesized. Genes related to the *MEG* gene were discovered in other plant species. We showed that the SlMEG2 peptide selected for the synthesis of the γ-core was suppressed by infection with *F. oxysporum* [11]. To test whether SlMEG2 possesses antimicrobial properties, in this work, we checked the antimicrobial activity of the peptide corresponding to the γ-core of SlMEG2. Since data on the 3D structure of MEG peptides is unavailable at the moment, we do not know if it adopts a β-hairpin structure. However, the net charge of the SlMEG2-derived peptide is positive (+2), favoring interactions with the membranes of the pathogens. For the first time, we discovered that the MEG-derived peptide displays antimicrobial activity towards several pathogens (*C. neoformans, C. michiganensis, B. cinerea* and *B. sorokiniana*) at a high peptide concentration, but is inactive against *Fusarium* species that may explain its suppression by *F. oxysporum* infection. These results support the existence of defense functions for MEG peptides.

Analyzing the predicted 3D structure of the CRP-derived peptides, we did not find any correlation between the predicted spatial structure of peptides and their antimicrobial activity. The most potent defensin- and snakin-derived peptides display either α-helical or random-coil conformation. However, similar 3D structures were predicted for the least active LTP-derived peptides. The factor that seems important for the antimicrobial activity of short peptides is the charge of the peptide, which is much higher in the peptides with pronounced antimicrobial properties than in the virtually inactive peptides.

The discovery of antimicrobial activity in the CRP peptides responsive to *F. oxysporum* infection and/or resistance inducers points to their role in innate and induced immunity mechanisms in tomato.

To gain an insight into the mode of action of the most active peptides, γ_58-74_SlDEFL4 and γ_89-106_SlSN9, were selected. Staining of *C. albicans* cells with propidium iodide in the presence of these peptides demonstrated that the peptides induce permeabilization of the fungal membranes. Accordingly, fungal cell death occurs at least partially due to membrane disruption. The interaction of the peptides with yet unknown intracellular targets cannot be excluded either and will be explored in our future studies.

The most active peptides discovered in this work will be further studied in detail to explore their stability, toxicity to human cells and biodegradability in nature to evaluate their ecological impact. All these future studies are vital for practical use of the peptides in either medicine or agriculture as biofungicides.

## 5. Conclusions

In this work, synthetic oligopeptide fragments corresponding to the γ-core regions of the selected CRP peptides belonging to the DEFL, snakin, nsLTP and MEG families were assayed in vitro against important human and plant pathogens. We discovered the antimicrobial activity in the peptides derived from two DEFLs (γ_58-74_SlDEFL2 and γ_58-74_SlDEFL4), three snakins (γ_48-65_SlSN2, γ_89-106_SlSN9 and γ_47-64_SlSN10), one nsLTP (γ_70-86_SlLTPg2.8) and one MEG (γ_92-104_SlMEG2), demonstrating their direct involvement in defense reactions in tomato. Interestingly, the MEG-derived peptide γ_92-104_SlMEG2 appeared active against the yeast-like fungus *C. neoformans* and the gram-positive bacteria *C. michiganensis,* which disclosed a novel, previously unknown defense function for the MEG peptides. Quite unexpectedly, the CRP-derived short peptides appeared more active not against plant pathogenic fungi, but against the gram-positive bacterium *C. michiganensis* that causes bacterial wilt—opening up new possibilities for their use in agriculture to control this dangerous disease. Furthermore, high inhibitory potency of short oligopeptides was demonstrated against the yeast *C. neoformans,* which causes serious diseases in humans, making these short peptide molecules promising candidates for the development of next-generation pharmaceuticals.

## Figures and Tables

**Table 1:** Physicochemical properties of the synthetic peptides.

Peptide Name	Peptide Property							
	Amino Acid Sequence	Length, aa Residues	Molecular Weight, Da	pI	Net Charge at pH 7	Aliphatic Index	GRAVY Index	Boman Index
γ_58-74_SlDEFL2	FSGGDCRGFRRRCFCTR	17	2024.33	10.39	+4	0.00	−0.753	4.33
γ_58-74_SlDEFL4	FTGGNCRGFRRRCFCTR	17	2037.37	11.40	+5	0.00	−0.747	4.16
γ_48-65_SlSN2	GACAARCRLSSRPRLCHR	18	2013.39	11.40	+5	60.00	−0.489	3.66
γ_89-106_SlSN9	GLCKYRCSLHSRPNVCFR	18	2139.54	9.61	+4	59.44	−0.383	2.6
γ_47-64_SlSN10	GSCKTRCSKSSRQNLCNR	18	2028.31	10.14	+5	21.67	−1.378	4.5
γ_56-72_SlLTPg2.4	SGCCTQLGTVVKNNPEC	17	1752.99	5.71	0	57.06	−0.165	1.23
γ_5__3__-__69_SlLTPg2.5	SGCCTQLSTVVDEKSEC	17	1788.98	4.14	−2	57.06	−0.141	1.82
γ_70__-__86_SlLTPg2.8	KGCCPEIAGLLDSNPIC	17	1733.05	4.37	−1	97.65	0.394	0.22
γ_92-104_SlMEG2	RPFGYCSFTPKTC	13	1506.76	8.90	+2	0.00	−0.377	1.51

**Table 2:** Antimicrobial activity of synthetic peptides *.

Pathogen	IC_50_, μM				
	γ_58-74_SlDEFL2	γ_58-74_SlDEFL4	γ_48-65_SlSN2	γ_89-106_SlSN9	γ_47-64_SlSN10
*Cryptococcus neoformans*	11.5 ± 2.5	8.1 ± 2.4	4.2 ± 0.6	5.1 ± 1.2	−
*Clavibacter michiganensis*	19.8 ± 2.5	21.5 ± 5.1	23.1 ± 2.7	24.0 ± 2.4	−
*Fusarium culmorum*	44.8 ± 4.0	42.3 ± 5.7	42.1 ± 6.5	42.4 ± 3.5	126.7 ± 8.5
*Fusarium oxysporum*	165.8 ± 18.4	124.8 ± 3.7	57.1 ± 11.6	−	43.8 ± 6.8
*Fusarium solani*	−	−	47.5 ± 2.0	138.8 ± 6.1	−
*Fusarium verticillioides*	−	−	152.0 ± 7.7	99.8 ± 10.0	−

* Mean values ± SD are presented; *C. neoformans* and *C. michiganensis* were incubated with peptides for 24 h and *Fusarium* species, for 38 h; “−“ not determined.
